# ST2 levels and neurodegenerative diseases: is this a significant relation?

**DOI:** 10.1097/MS9.0000000000001939

**Published:** 2024-03-12

**Authors:** Himanshu Arora, Binish Javed, L V Simhachalam Kutikuppala, Mayuri Chaurasia, Kaarvi Khullar, Shreevikaa Kannan, Varshitha Golla

**Affiliations:** a Department of General Medicine, Netaji Subhash Chandra Bose Subharti Medical College, Meerut, Uttar Pradesh; b Atal Bihari Vajpayee Institute of Medical Sciences & Dr. Ram Manohar Lohia Hospital, New Delhi; c Department of General Surgery, Dr NTR University of Health Sciences, Vijayawada, Andhra Pradesh; d National Institute of Medical Sciences and Research, Jaipur, Rajasthan; e Government Medical College, Gondia, Maharashtra, India; f Department of General Medicine Tbilisi State Medical University, Tbilisi, Georgia; g Department of General Medicine, International School of Medicine (ISM), Bishkek, Kyrgyzstan

**Keywords:** central nervous system (CNS), IL-33, soluble ST2 levels, neurodegenerative disorders

## Abstract

Interleukin-33 (IL-33), belonging to the interleukin-1 cytokine family, has a decoy receptor soluble ST2 (sST2). IL-33 is found in oligodendrocytes and astrocytes and is involved in central nervous system healing and repair, whereas ST2 is found in microglia and astrocytes. Some studies have found a link between changes in the IL-33/ST2 pathway and neurodegenerative disorders. This review article investigates the relationship between the interleukin-33 (IL-33)/ST2 pathway and neurodegenerative disorders. It was discovered that soluble st2 levels were increased. Furthermore, IL-33 levels were found to be lower in many neurodegenerative diseases such as Alzheimer’s and amyotrophic lateral sclerosis (ALS). The association with other disorders, such as ankylosing spondylitis, multiple sclerosis, and systemic lupus erythematosus (SLE), was also observed. Various studies suggest that ST2/IL-33 signalling may be pivotal in the disease modulation of neurodegenerative disorders. The serum sST2 level test can be useful in determining the inflammatory status and severity of illness in many neurodegenerative disorders. In this review, we will discuss recent findings concerning the interleukin-33 (IL-33)/ST2 pathway and its role in the diagnosis and treatment of diseases with neurodegeneration.

## Introduction

Highlights

- Interleukin-33 (IL-33) levels were found to be lower in many neurodegenerative diseases such as Alzheimer’s and amyotrophic lateral sclerosis (ALS).
- The findings from various studies suggest that ST2/IL-33 signalling may be pivotal in the disease modulation of neurodegenerative disorders.
- The serum sST2 level test can be useful in determining the inflammatory status and severity of illness in many neurodegenerative disorders.
- There are currently no clinical studies employing IL-33/ST2 signalling to treat central nervous system (CNS) disorders.

Neurodegenerative illnesses are conditions marked by a gradual loss of neurons in the brain and other peripheral organs, along with the accumulation of proteins exhibiting changed physicochemical characteristics. For most brain-related illnesses, neurodegeneration has been found to be the primary pathophysiological alteration. Neuron loss is the main feature of diseases with neurodegeneration, and the most prevalent neurodegenerative illnesses are Parkinson’s and Alzheimer’s diseases (AD)^1^. ST2 was identified as a member of the Interleukin-1 (IL-1) family as an orphan receptor in the year 1989. Further studies showed that ST2 has a significant role in inflammatory pathways involving mast cells and type 2 CD4+ T-helper cells. ST2 was identified as a particular cellular marker that produces Th2-associated cytokines and distinguishes Th2 T-cells from Th1 T-cells^2^. Recently, interleukin-33 (IL-33) was identified to be a ligand of ST2 ligand, which offered fresh perspectives on ST2 signalling. IL-33 has been found to be a key mediator in several inflammatory illnesses^3^. The innate immune response is crucially mediated by IL-33, controlling the immune cell infiltration and activation. Myeloid differentiation factor 88 (MyD88) and NF-B are involved in the intracellular cascade which is initiated when IL-33 binds to a heterodimeric receptor complex made up of ST2 and IL-1RAcP. This pathway leads to the activation of macrophages, type 2 T-helper cells, neutrophils, and mast cells^4,5^. ST2 is expressed predominantly by microglia and astrocytes^6^. Both microglia and astrocytes express IL-33 receptors and respond by proliferating and generating IL-1β and TNF-α, making IL-33 particularly pro-inflammatory in the central nervous system (CNS)^7,8^.

Presently, AD is the most common type of dementia among the older population^9^. Worldwide, ~47 million people have Alzheimer’s or related dementia, and this figure is estimated to reach about 131 million by 2050^10,11^. The major causes of this disease are plaques (chemical deposits of beta-amyloid formed in between nerve cells) and tau proteins (lead to the formation of tangles and disrupt the transport system inside the neuron) that cause damage and death of neurons^11,12^. Genetic research has shown to link the 3 single nucleotide polymorphisms in IL-33 to a lower risk of developing AD^12^. As a mediator of inflammatory molecules, IL-33 has also been linked to the pathogenesis of Alzheimer’s disease. The increased expression of IL-33 in astrocytes and glial cells present in the CNS has been linked to pathogen-associated molecular patterns (PAMPs). Its expression is also amplified by mast cells, which leads to the induction of several immune effectors in CNS glia in particular arginase I, CCL17, TNF- α, and CCL11^13^. The most common neurological disability, multiple sclerosis (MS), is an autoimmune-mediated condition that impacts the CNS, causing neurodegeneration. Since MS is characterised by immune-mediated demyelination of axons, the involvement of IL-33 is of special interest since IL-33 has the capacity to modify not only the immune system but also the CNS and, as a result, its effect on the disease pathology^14^. The primary causes of myelin sheath destruction are focal T-lymphocytic and macrophage infiltrations and oligodendrocyte death that lead to the formation of CNS plaques made up of inflammatory cells and their by-products. It also causes the production of demyelinated and transected axons and astrogliosis in both white and grey matter^15,16^. In patients with MS, IL-33 is elevated in CNS and peripheral tissues, especially in the white matter and plaque region of the brain^17^. Systemic lupus erythematosus (SLE) is an autoimmune connective tissue disease primarily affecting young women of reproductive age. It is characterised by persistent and abnormal activation of the immune system against self-antigens, which eventually results in the development of autoantibodies and immune complexes (ICs), causing further harm to several organs, namely kidneys, joints, lungs, brain, and skin^18^. IL-33 levels were shown to be considerably higher in the serum of SLE patients compared to healthy controls in various investigations^19^. ALS then causes fast-progressing skeletal muscular paralysis, which ultimately results in death from respiratory failure, generally three to five years after the illness first manifests. The degradation of motoneuron in ALS is significantly influenced by the astrocytic production of pro-inflammatory mediators^20–23^. It has been found that ALS patients had lower levels of IL-33 and higher amounts of soluble ST2^24^.

This review goes on to outline the research and data that link the IL-33/ST2 pathway to the pathophysiology of several diseases with neurodegeneration, including AD, MS, ALS, and SLE. In both innate and adaptive immunity, it appears that IL-33 functions as a crucial inducer and regulator of gene transcriptions and cytokines, either as an intracellular or extracellular cytokine. Additionally, a thorough analysis of ST2’s effects on different types of nervous system cells has been done. Future possibilities, innovative therapy techniques, and their implications for future investigations have also been discussed.

## Methodology

A literature search was conducted to identify articles and studies related to the relationship between ST2 levels and Neurodegenerative disorders. This review did not involve a systematic search of articles. Instead, we focused on including the most relevant and current articles based on our selection criteria. The databases searched were PubMed and Google Scholar. The search was conducted using the following keywords and their combinations: “ST2 levels and Amyotrophic Lateral Sclerosis”, “ST2 levels and Ankylosing spondylitis”, “ST2 levels and Systemic Lupus Erythematosus”, “ST2 levels and Alzheimer’s Disease”, “IL-33/ST2 Pathway Functions in Neurodegenerative Diseases”, “IL-33/ST2 signalling pathway and its effect on various cells of the nervous system”, and “Role of IL-33/ST2 on myelination”. We categorised and qualitatively described the studies into thematic sections based on the relationship between ST2 levels and various neurodegenerative disorders and their role in diagnosis. Inclusion criteria for the articles were: (1) studies or articles that focused on the studies investigating the relationship between ST2 levels and neurodegenerative disorders, (2) studies or articles published in English, (3) studies or articles published from the earliest available date to May 2023, and (5) studies or articles with full-text availability. The findings of this review provide insights into the current state of research on the relationship between ST2 levels and neurodegenerative disorders. The narrative synthesis approach used in this review allows for the identification of patterns and consistencies across the included studies. This analysis may reveal common findings, trends, or potential associations between ST2 levels and neurodegenerative disorders. Limitations of this research include potential publication and language bias, reliance on existing studies, limited generalisability, and conflicting evidence.

### Amyotrophic lateral sclerosis

Amyotrophic lateral sclerosis (ALS) is a neurodegenerative motor neuron disease that affects both upper and lower motor neurons. In most cases, the disease’s effects on the respiratory muscles restrict survival to 2–4 years following disease onset, manifesting as a relentlessly progressing muscle atrophy and weakening^25^. In a recent study, ELISA was used to measure the levels of IL-33 and s ST2.IL-33 levels in ALS patients were substantially lower than in healthy controls, although sST2 levels were significantly higher. Lower IL-33 levels might be caused by caspases that apoptotic cells release that break down IL-33. However, sST2, which functions as an IL-33 receptor, may be having a negative impact on IL-33 levels. sST2 levels may indicate inflammation in ALS^26^. Numerous additional studies also found that levels of Il-33 were lower and levels of St2 were higher in ALS patients^27,28^. A transgenic mouse research found that the Th2-type cytokine IL-33 delayed the onset of the disease in female ALS mice. Female mouse lymph nodes and spleens produced fewer T lymphocytes as a result of IL-33. In female mice, IL-33 reduced astrocytic activation of the spinal cord. The medication had no effect on the male mice^29,30^. Treatment with IL-33 improved symptoms in SOD1G93A transgenic mice, proving that it is an important downstream modulator of ALS development. Considering this, it may be said that IL-33 and ST2 levels are crucial for ALS care^31,32^.

### Systemic lupus erythematosus

**Figure 1:**
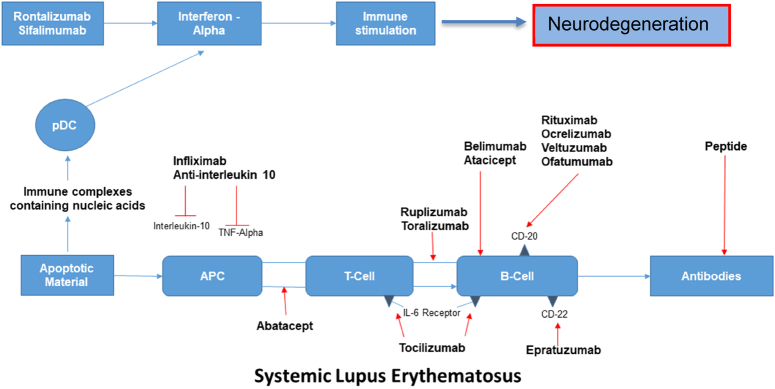
Mechanism underlying systemic lupus erythematosus.

A multisystem autoimmune illness with high morbidity and death is SLE. The production of pathogenic autoantibodies that damage tissue in several ways is triggered by the loss of immunological tolerance to self-antigens, which is influenced by genetic, immunological, endocrine, and environmental variables^33,34,35^. Serum sST2 levels in SLE patients were shown to be changeable and linked with disease activity, indicating a potential role as a substitute marker of disease activity. In one investigation, patients with active SLE had serum sST2 levels that were considerably greater than those with inactive SLE (0.51 (0.18) ng/ml vs. 0.42 (0.08) ng/ml). as well as healthy controls [0.36 (0.13) ng/ml] (*P* = 0.006) (*P* < 0.001)^36^. In another study, sST2 was discovered to be a stand-in marker of disease activity and consequences from nephritis. In SLE patients, IL-33 levels were also elevated^19^. Systemic Lupus Erythematosus Disease Activity Index (SLEDAI) results demonstrated a correlation between IL-33 concentrations and disease activity^37^. Extracellular IL-33 complexes with NET levels were observed to be increased in the blood, skin, and kidney biopsies from SLE patients, and this correlated with disease activity. Ex-vivo testing demonstrated that SLE patients’ neutrophils produced IL-33-decorated NETs, which in turn prompted DCs to launch a powerful type I IFN response by activating ST2^38^. In contrast to SLE patients without renal involvement, Moreau and colleagues found that patients with lupus nephritis had statistically higher levels of sST2 serum^3^. Patients in a different study who had more severe SLE also had greater levels of sST2, CXCL10, and hs-troponin (SLEDAI). The SLE damage index showed that patients with higher sST2 and CXCL10 levels had more disease damage^39^. IL-33 functions in SLE as an immunoregulatory, a stimulator of tissue healing, and a pro-inflammatory alarmin. In the next years, it may be effective to treat SLE by focusing on the IL-33/ST2 axis. (Fig. 1)^40^.

### Alzheimer’s disease

**Figure 2:**
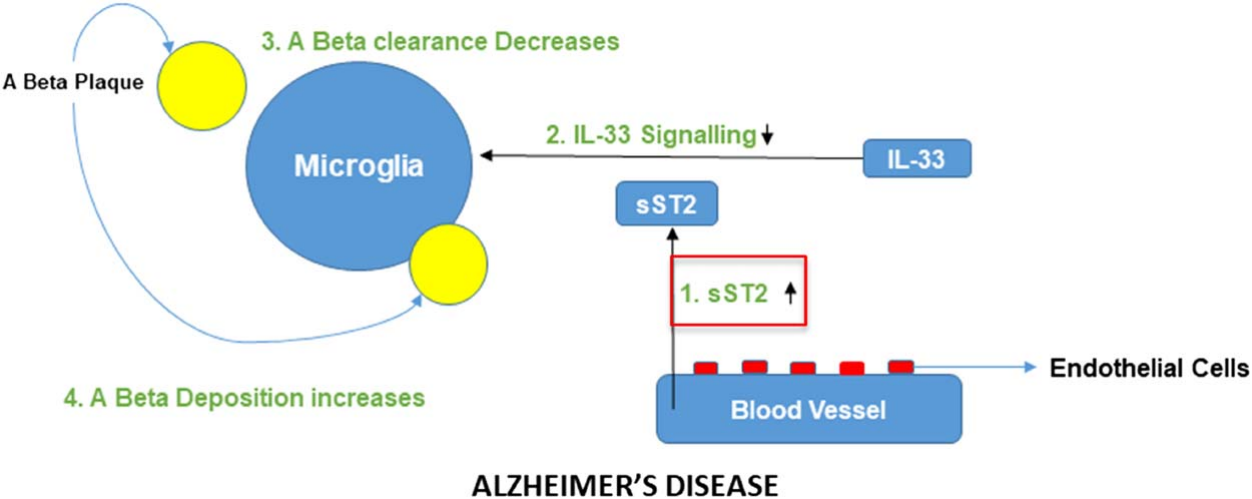
Mechanism underlying Alzheimer’s disease.

The most prevalent neurodegenerative condition and the main cause of death in older individuals is AD. Microglial A-clearance capacity is impacted by changes in sST2 levels in the brain milieu, which influences AD risk and related pathological alterations. sST2 modulates microglial activity^41^. A potential treatment target for AD is sST2. Firstly, because endothelial cells are the primary ones that express sST2, sST2 expression can be altered in ways that may not need crossing the blood-brain barrier. The possibility of using sST2 in early intervention techniques is suggested by the fact that sST2 levels are elevated in people with MCI or early-stage AD. A common variant associated with AD is the allele rs1921622 A. By focusing on this genetic variant, sST2 manipulations could be developed for specific subgroups of people with high sST2 levels (for instance, females who carry APOE-4 but not the rs1921622 A allele, who account for 6.2–12.2% of people with AD). This would allow for patient stratification and precision medicine. (Fig. 2)^42^.

### IL-33/ST2 pathway functions in diseases with neurodegeneration

The involvement of pathway has been implicated in various illnesses for example graft-versus-host illness and conditions like inflammatory bowel disease that is active, acute cardiac and small bowel transplant allograft rejection, gut mucosal damage brought on by viral infection, pulmonary illnesses, and heart disease, among others, in patients. However, other analyses have shown that the pathway is concerned with neurodegenerative illnesses, notably those that affect the CNS. Both ST2L/IL-1RAcP and IL-33 are widely formulated in the brain’s nerve tissue, although ST2L/IL-1RAcP is only expressed by microglia, astrocytes, and neurons, whereas IL-33 is expressed by endothelial cells, astrocytes. neurons and oligodendrocytes^43–45^. It was also recognised that IL-33 mRNA was substantially expressed in the cerebrum and spinal cord of rodents^46^. When IL-33 is introduced to microglia, it triggers the production of several pro-inflammatory cytokines, including TNF-*α*, IL-1*β*, and IL-10, as well as chemokines, such as CCL2, CCL3, CCL5, and CXCL10 and molecules associated with oxidative stress, such as nitric oxide (NO) and nitric oxide synthase^45^. As described in Table 1, the well-defined and extremely important involvement of IL-33/ST2 in neurodegenerative illnesses is strongly proven in Alzheimer’s disease and multiple sclerosis.

**Table 1:** Pathophysiological findings of various diseases with neurodegeneration

Disorder	Findings	References
Alzheimer’s disease	i. PAMPs induced IL-33 mRNA and p protein production in the CNS glia.	^47^
	ii. IL-33 amplified and stimulated the mast cells directly by inducing the arginase I, CCL17, CCL11, and TNF-α in CNS glia.	^48^
	iii. IL-33 indicated that it is reduced in the tissues of the brains.	
	iv. IL-33 decreases the secretion of Aβ40 peptides when overexpressed.	
	v. The production of CCL2, CCL3, CCL5, CXCL10, nitric oxide, and phagocytic activity of microglia was enhanced by IL-33	
	vi. IL-33 induces proliferation of microglia and it also stimulates the production of IL-1β, TNF-α, and IL-10.	^45^
	vii. IL-33 and ST2 expression was highly expressed in βA plaque, NFTs, and glial cells in the AD brains.	^49^
	viii. In the nerve tissues of the IL-33 and ST2+ cells were increased.	
	ix. Decrease in the soluble βA levels and plaque accumulation by IL-33.	^14^
	x. IL-33 decreases IL-1β, IL-6, & NLRP3 and its modulation is polarising macrophages in the nerve tissues of the brain	
Multiple sclerosis	i. IL-33 was found to be highly expressed in peripheral blood and CNS and plague areas in MS brains.	^50,51^
	ii. The plasma level of IL-33 was increased, when compared with the control.	^51^
	iii. There was a decrease in plasma level IL-33 in MS patients treated with IFN-β-1a.	
	iv. Highly expressed intracellular IL-33 and OAS1, GATA3, and PMAIP1.	^53^
	v. In RRMS patients IL-33 and HDAC were highly induced.	
	vi. Gene regulation in RRMS might be done by IL-33.	
	vii. In patients with chronic and acute MS, the IL-33 and ST2 expressions were enhanced as compared to the controls.	
	viii. The CNS myelination might be suppressed by IL-33.	

AD, Alzheimer’s diseases; CNS, central nervous system; HDAC, HistoneDeacetylase; IL, interleukin; NFT, Neurofibrillary tangle; RRMS, RelapsingremittingMultiple Sclerosis; TNF, Tumornecrosis factor.

### IL-33/ST2 signalling pathway and its effect on various cells of the nervous system

The IL-33/ST2 signalling pathway is crucial in stimulating cells associated with our immune system and nervous system. IL-33 interacts through a heterodimer composed of the IL-1 receptor-related protein ST2 and the IL-1 receptor accessory protein. The ST2 receptor for IL-33 signals has been found to be expressed in a variety of tissues, which include stressed cardiac myocardial cells and airway epithelia^14^. Both ST2L and sST2 are expressed in astrocytes and microglial cells, whereas sST2 is only expressed in brain endothelial cells (2). In contrast, the IL-33 cytokine is expressed by several CNS cells, including neurons, astrocytes, oligodendrocytes, and microglial cells^8^.

Astrocytes are thought to be cells that express the entire IL-33 sequence. It is also abundant in the spinal cord and brain, with the greatest concentration found in white matter areas such as the corpus callosum^8,52^. However, it was discovered that IL-33 is expressed only in the CNS during late embryogenesis and then disappears as the neonate’s brain develops into an adult brain^52^.

Apart from the CNS, it is found in the stomach, lungs, skin, and lymph nodes. IL-33 can regulate both the immune system and the CNS system^54^. The signalling pathway performs a plethora of functions in inflammatory and infectious diseases, most often acting as a dual mediator of both tissue repair and inflammatory diseases. The IL-33/ST2 signalling pathway activates T-helper 2 cells, Basophils, and Mast cells, causing them to produce TH2 cytokines and acting as a powerful mediator in any allergic reaction. However, IL-33 and its ability to induce the proliferation of pro-inflammatory cytokines is dose-dependent. Dendritic cell function in the CNS is regulated by IL-33^55^.

Microglia, the primary cells of innate immunity, increase in number when treated with IL-33. The FCM essay showed a marked increase in the activity of microglial cells after being treated with IL-33 for 24 h at a fixed concentration of 0.1 ng/ml or higher, that is phagocytic activity increased by multiple folds. It was observed that there is a feedback loop that influences glial activity. The role of IL-33 in inflammatory diseases in the CNS is marked by the demyelination of axons, which is moderated by the body’s immune system^55^.

### Role of IL-33/ST2 on myelination

The IL-33/ST2 axis is important in the pathogenesis of many CNS diseases, including neurodegenerative diseases, cerebrovascular diseases, infectious diseases, traumatic CNS injury, chronic pain, and others^50^. Patients with mild cognitive deformity have higher serum levels of soluble ST2 (sST2), a decoy receptor for interleukin (IL)-33^51^. It was discovered that stimulating the IL-33/ST2 signalling pathway reduces memory-related symptoms in Alzheimer’s Disease, a very common neurodegenerative disorder characterised by memory deficit manifestations caused by the accumulation of B amyloid plaque. According to a study conducted on transgenic mouse models, IL-33 administration restores synaptic plasticity and promotes the phagocytic activity of microglial cells, which ultimately reduces the soluble B amyloid plaque^54^.

Furthermore, the critical role of IL-33 as an active component causes aberrant local and systemic damage, as seen in a variety of inflammatory disorders and immune-mediated pathological conditions such as multiple sclerosis (MS), systemic lupus erythematosus (SLE), psoriasis, Sjogren’s syndrome, inflammatory bowel disease (IBD), and others. In autoimmune disease, the IL-33/ST2 axis can increase pro-inflammatory cytokine release; however, in some metabolic diseases, such as type 1 diabetes, IL-33 can be considered an anti-inflammatory cytokine^56^. In a mouse model of traumatic brain injury, IL-33/ST2L signalling provides neuroprotection by inhibiting autophagy, endoplasmic reticulum stress, and apoptosis. The increase in IL-33 and decrease in ST2L levels in the injured cortex were first observed 24 h after the TBI. IL-33 treatment also improved TBI-induced brain water content, motor function outcome, and spatial learning and memory deficits. IL-33/ST2 signalling reduces TBI-induced brain oedema, motor function result, spatial learning, and memory problems are caused, at least in part, by a system including autophagy suppression, ER stress, apoptosis, and neuroinflammation. The TBI group showed an increase in IL-33 expression, while the TBI group showed a decrease in ST2L expression. Exogenous IL-33 or SAL alone significantly reduced ST2L protein expression as compared to the TBI group^57^. However, when SAL and IL-33 were administered together, the amount of ST2L expression rose when compared to IL-33 or SAL alone. TBI dramatically increased IL-33 expression, and IL-33 staining was largely found in the nucleus. Aside from minimising cerebral oedema, on days 1–7 after TBI, IL-33 therapy significantly improved motor functional outcomes. The IL-33/ST2 signalling pathway reduces endoplasmic reticulum stress (ERS) following TBI^58^.

## Conclusion

In the current review, the ST2 receptor, which was discovered as an orphan receptor for the IL-1 family, has been discussed. The studies that have been included in the review suggest that IL-33 functions either as an intracellular or extracellular cytokine in innate and adaptive immunity as a crucial inducer and regulator of gene transcriptions and cytokines. Therefore, further research is needed to understand these pathways fully since they may serve as advantageous targets in CNS illnesses as new biomarkers, therapeutic targets, or even interventional goals. Most current research links local lesions and/or lymphatic tissues’ anti-inflammatory milieu to increased IL-33 production caused by damaged CNS (spleen and LNs). However, IL-33 is crucial for maintaining tissue homoeostasis, responding to damage and inflammation, and managing gut flora. Currently, there are no clinical studies employing IL-33/ST2 signalling to treat CNS disorders. Future research needs to be conducted into utilising the IL-33/ST2 pathway as a therapeutic target for disorders of the central nervous system. From bench to bedside for IL-33/ST2 treatment in CNS illnesses, there is still a long way to go.

## Ethics approval

Ethics approval was not required for this review.

## Consent

Informed consent was not required for this review

## Source of funding

Not applicable.

## Conflicts of interest disclosure

Not applicable.

## Research registration unique identifying number (UIN)

Not applicable.

## Guarantor

Varshitha Golla.

## Data availability statement

Not applicable.

## Provenance and peer review

Not commissioned, externally peer-reviewed.
